# Classifying Unsafe Health and Care Behaviors in the Context of Just Culture: A Comparative Video-Based Vignette Study

**DOI:** 10.3390/healthcare14193275

**Published:** 2026-10-02

**Authors:** Susana Lorenzo, Eva Gil-Hernández, Irene Carrillo, Cristina Navarro, Pedro Lozano Gago, Marta Macías Maroto, Elvira Eva Moreno Campoy, Paloma Trillo López, Elena Corpas Nogales, Ángel Cobos-Vargas, Mª Del Carmen Jiménez Estrada, Pastora Pérez-Pérez, María Teresa Gea-Velázquez de Castro, Mercedes Arencibia-Jiménez, Alberto Asencio, Juan Molina Ribera, Jesús María Aranaz-Andres, Diego San Jose-Saras, José J. Mira

**Affiliations:** 1 Hospital Universitario Fundación Alcorcón, 28922 Alcorcón, Spain; slorenzom@salud.madrid.org; 2 ATENEA Research Group, Foundation for the Promotion of Health and Biomedical Research of Valencia Region (FISABIO), 03550 Sant Joan d’Alacant, Spain; eva.gilh@umh.es (E.G.-H.); cristina.navarro@fisabio.es (C.N.); 3 Health Psychology Department, Miguel Hernández University, 03202 Elche, Spain; icarrillo@umh.es; 4 Multiprofessional Teaching Unit for Family and Community Health of Alicante, 03009 Alicante, Spain; pedro.lozanogago@gmail.com; 5 Príncipe de Asturias University Hospital, 28805 Alcalá de Henares, Spain; marta.maciasm@salud.madrid.org; 6 Andalusian Patient Safety Strategy, Ministry of Health, Presidency and Emergencies, Government of Andalusia, 41020 Seville, Spain; eeva.moreno.sspa@juntadeandalucia.es (E.E.M.C.); paloma.trillo.sspa@juntadeandalucia.es (P.T.L.); elena.corpas.easp@juntadeandalucia.es (E.C.N.); 7 Andalusian School of Public Health, Ministry of Health, Presidency and Emergencies, Government of Andalusia, 18011 Granada, Spain; 8 Intensive Care Unit, San Cecilio University Hospital, 18016 Granada, Spain; angel.cobos.sspa@juntadeandalucia.es; 9 ibs.GRANADA Health Research Institute, 18071 Granada, Spain; 10 San Juan de Dios del Aljarafe Hospital, 41930 Bormujos, Spain; mariacarmen.jimenez@sjd.es; 11 Territorial Unit II, Province of San Juan de Dios of Spain, 41005 Seville, Spain; pasperper@gmail.com; 12 Department of Public Health, History of Science and Gynaecology, University Miguel Hernández of Elche, 03550 Alicante, Spain; gea_mte@gva.es; 13 Department of Preventive Medicine, Sant Joan d’Alacant University Hospital, 03550 Sant Joan d’Alacant, Spain; arencibia_mer@gva.es; 14 Primary Care Centre of Mutxamel, Alicante-Sant Joan Health District, 03110 Alicante, Spain; alberto.asencio.aznar@gmail.com; 15 Primary Care Directorate, Alicante-Sant Joan Health District, 03550 Sant Joan d’Alacant, Spain; molina_juarib@gva.es; 16 Department of Preventive Medicine and Public Health, Ramón y Cajal University Hospital, IRYCIS, 28034 Madrid, Spain; jesusmaria.aranaz@salud.madrid.org (J.M.A.-A.); diego.sanjose@salud.madrid.org (D.S.J.-S.); 17 Faculty of Health Sciences, International University of La Rioja, 26006 Logroño, Spain; 18 CIBER of Epidemiology and Public Health (CIBERESP), 28029 Madrid, Spain; 19 Provincial-Pla Hospital, Alicante-Sant Joan Health District, 03013 Alicante, Spain

**Keywords:** Just Culture, patient safety, accountability, healthcare organizations, public trust, video vignettes, restorative justice

## Abstract

**Highlights:**

**What are the main findings?**

- Video vignettes were used to explore how groups judge unsafe healthcare behaviors.
- At-risk behavior accounted for 40.5% of valid classifications.

**What are the implications of the main findings?**

- Exact concordance with the prespecified reference classification was 48.4%.
- Variability in interpreting unsafe behaviors may be relevant to Just Culture implementation.

**Abstract:**

**Background**: Just Culture requires organizations to distinguish human mistakes from unsafe behavior while maintaining transparency, accountability and trust. However, little is known about how different stakeholders interpret these boundaries in realistic healthcare situations. This study aimed to explore how unsafe healthcare behaviors are classified by professionals and community and public-opinion stakeholders within the framework of Just Culture. **Methods**: This structured judgment study used standardized video vignettes across primary care, hospital, and nursing home settings. A purposive and convenience sample of 449 healthcare professionals, managers, and community/public representatives classified behaviors as honest error, at-risk behavior, or reckless behavior and provided written justifications. Concordance with a prespecified study-defined reference classification was examined using mixed-effects logistic regression. **Results**: Across 2976 classification opportunities, 2610 were analyzable within the three predefined categories. At-risk behavior was most frequent (40.5%), followed by honest error (33.5%) and reckless behavior (25.9%). Exact concordance with the prespecified reference classification was 48.4% and was highest for reckless behavior (67.4%). Participants’ justifications for their classification choices centered on intention, contextual pressure, foreseeability, omitted safety barriers, professional duty, and relational expectations. **Conclusions**: Participants generally distinguished human fallibility from the omission of available safeguards, but many behaviors were located in an intermediate zone of at-risk conduct rather than clear error or recklessness. The observed variability in interpreting error, risk, and responsibility, particularly around at-risk behavior, may be relevant to the implementation of Just Culture.

## 1. Introduction

All healthcare professionals, regardless of their qualifications or experience, are susceptible to error. Explicit recognition of human fallibility is essential for improving patient safety [1], since it enables the design of safer systems, procedures, technologies, and working environments for both patients and healthcare workers [2]. Contemporary patient safety theory recognizes that errors are rarely caused by isolated individual failures and more commonly emerge from the interaction between human limitations and the conditions under which care is delivered [3,4].

Cognitive overload, fatigue, stress, interruptions, multitasking, workload pressure, communication failures, and poorly designed systems are well-established contributors to unsafe acts in healthcare [5]. Under demanding clinical conditions, even experienced professionals may unintentionally deviate from expected performance.

The belief that competent professionals should not make mistakes encourages retrospective judgments centered on individual responsibility rather than on the analysis of contributing factors and system vulnerabilities [2,6]. This perspective may distort incident investigation, discourage reporting, hinder transparency after adverse events, and negatively affect both patients and healthcare professionals [1]. Fear of blame, disciplinary consequences, reputational damage, or legal exposure remains an important barrier to open communication and learning after safety incidents [7,8].

In response to these limitations, contemporary patient safety approaches increasingly seek to balance learning and accountability after safety incidents [9,10]. A central challenge for Just Culture is how error and individual responsibility are conceptualized. Distinguishing honest error, which arises from human fallibility within complex systems, from at-risk and reckless behaviors is crucial for promoting transparency, reporting, and organizational learning while ensuring proportionate accountability. Honest error generally calls for support, learning, and system redesign, whereas at-risk behaviors involve unsafe actions in which expected safeguards are omitted, often because risk is underestimated or normalized rather than deliberately accepted [11]. Reckless behaviors imply conscious disregard of a substantial and unjustifiable risk and may require corrective or disciplinary responses [12,13]. This distinction is essential because organizational responses should differ according to the nature of the behavior and the surrounding context.

Once behavior and responsibility have been interpreted, the focus extends beyond classification and proportionate accountability to acknowledging harm, restoring trust, supporting affected patients and professionals, and facilitating organizational and relational repair after adverse events [14]. In this context, the way different actors involved in the care process conceptualize error, risk and responsibility is central to building a safety-generating culture. Such conceptualizations influence how incidents are interpreted, whether they are openly reported and discussed, and how organizations respond after harm.

Implementation of restorative approaches may be particularly challenging in continental European civil-law systems, such as Spain, where the legal context differs from Anglo-American settings. In such environments, concerns about personal accountability and legal consequences may create tension between transparency, learning, and professional self-protection [2,15]. Attributional and outcome biases may reinforce this tension by favoring interpretations of unsafe behavior in terms of individual responsibility rather than the interaction between human fallibility and system conditions [16,17], potentially hindering learning-oriented and restorative responses.

The interpretation of unsafe behaviors remains complex and may be influenced by professional culture, organizational expectations, previous experiences, social values, and legal traditions [18,19]. Healthcare professionals may also share blame-oriented interpretations of unsafe behavior. However, their familiarity with patient safety principles, system factors, and risk-management strategies may lead them to interpret error and responsibility differently from patients, journalists, or the wider public [3,20]. Despite the growing implementation of Just Culture policies in healthcare organizations, limited evidence exists regarding how different professional and public stakeholder groups interpret the boundaries between honest error, at-risk behavior, and reckless behavior in realistic clinical situations.

The present study aimed to explore how healthcare professionals and community and public representatives classify unsafe healthcare behaviors within the framework of Just Culture. Specifically, the study examined how participants distinguished between honest error, at-risk behaviors, and reckless behavior across a series of standardized video-based clinical scenarios, and identified the contextual, professional, organizational, and ethical factors underlying these judgments.

## 2. Materials and Methods

### 2.1. Study Design

This study used a structured judgment design based on standardized video-based clinical vignettes that functioned as trigger films to elicit individual judgments about unsafe healthcare behaviors. Participants individually categorized the behaviors portrayed in each vignette as an honest error, at-risk behavior, or reckless behavior, and provided written explanations for the reasoning underlying their classifications using a structured response booklet. The audiovisual scenarios served as standardized elicitation stimuli, allowing participants exposed to a given vignette to evaluate an identical representation of the clinical situation while preserving contextual complexity. The simulated setting was chosen because the scenarios involved patient safety situations that could not be ethically or safely reproduced in routine clinical practice.

The study combined quantitative analysis of participants’ classifications, including concordance with a prespecified reference classification, and descriptive qualitative content analysis of their written explanations. Both types of data were collected concurrently through the same response booklet and were integrated during interpretation to examine not only how behaviors were classified but also the reasoning participants used to support their judgments.

### 2.2. Setting

The study was conducted between April 2025 and February 2026. Reporting followed the Strengthening the Reporting of Observational Studies in Epidemiology (STROBE) [21] statement for observational studies and was additionally informed by the GROVE guideline [22] for reporting vignette-based studies in healthcare settings.

The study protocol has been published elsewhere [23] and was registered at ClinicalTrials.gov (NCT06835517) on 23 January 2025.

### 2.3. Development of Video-Based Vignettes

Video-based vignette methods offer an opportunity to explore these judgments under standardized conditions while preserving contextual realism. In this study the vignettes were used as trigger films [24]. By presenting participants with plausible healthcare scenarios involving ambiguity, workload, interruptions, system pressure, and discretionary decision-making, vignette studies may help identify how individuals interpret unsafe behaviors and which factors influence their classifications [25].

Seven short video-based clinical vignettes were developed to assess how participants classified unsafe healthcare behaviors within a Just Culture framework. The scenarios were designed to represent realistic patient safety situations occurring in primary care, hospital care, and nursing home settings. All scenarios were based on real clinical situations either directly experienced or reported by healthcare professionals, although identifying elements were modified to preserve anonymity and avoid recognition of specific patients, professionals, institutions, or events. Fifteen professionals from different specialties and care settings were asked to describe, without disclosing any personal data, safety incidents known to them through their professional experience. From these narratives, the cases that best represented situations suitable for analyzing whether the behavior reflected an honest error, at-risk behavior or reckless behavior were selected by the team. Based on this selection, scripts were prepared for the development of the videos. For each selected incident, the healthcare professional who contributed the original case also provided an initial interpretation of the relevant behavior or behaviors according to the three categories: honest error, at-risk behavior, or reckless behavior. These initial classifications were considered provisional and were subsequently examined during pilot testing before a final reference classification was established. The final selection of vignettes sought to maximize variation in contextual pressure, foreseeability, omitted safeguards, supervision demands, communication failures and ethical tension across healthcare settings.

To improve accessibility, standardization, and participant engagement, the scenarios were produced in a comic-style audiovisual format using fictional characters and anonymized clinical contexts while preserving clinical plausibility. Videos ranged from 2:40 to 4:29 min and were presented in a standardized format.

The vignettes were intentionally designed to portray professional behaviors potentially compatible with honest error, at-risk behavior, or reckless behavior under varying degrees of contextual pressure, ambiguity, workload, interruptions, multitasking, communication failures, system pressure, foreseeable risk, availability of safety barriers, and professional responsibility. Although all scenarios involved patient safety incidents, they differed in clinical context, supervision, communication demands, procedural complexity, and perceived preventability. Despite these differences, they were designed to represent comparable underlying behavioral and decision-making dimensions relevant to Just Culture.

None of the scenarios involved a fatal outcome. This was a deliberate design decision for this initial study to reduce the possibility that outcome severity would disproportionately influence judgments of responsibility and behavioral severity independently of the conduct itself.

During the development of the vignettes, the study team worked with a company specialized in the production of this type of video material. A detailed description of the scenarios and classification framework is provided in Appendix A, and the final vignettes are available at https://www.youtube.com/playlist?list=PL7t2xVITdKhjh5XBSGymS3HxLnWQJnsTC (accessed on 9 December 2025).

### 2.4. Pilot Testing and Refinement

Before the main study, six focus group sessions were conducted across four locations to evaluate the clarity, plausibility, realism, and interpretability of the preliminary video scenarios. A total of 60 healthcare professionals with diverse clinical backgrounds participated in the sessions. Women represented 61.7% of participants. Professional backgrounds included physicians (60.0%), nurses (23.3%), pharmacists (5.0%), and other healthcare providers (e.g., residents, physiotherapist, radiologic technologists) (11.7%). They had an average of 18.7 years of experience in patient safety activities. Eligible healthcare professionals were required to have at least five years of professional experience. Participants involved in the pilot testing were not included in the main study.

Participants reviewed the preliminary versions of the videos and identified inconsistencies, ambiguous elements, unrealistic aspects, or narrative problems that could affect interpretation of the behaviors represented. This classification exercise also served to examine the provisional classifications assigned during vignette development. Pilot participants first classified the behaviors individually according to the three categories and subsequently discussed the reasoning underlying their judgments. The research team reviewed the initial classification together with the judgments and reasoning obtained during pilot testing. Where the provisional classification and the pilot judgments converged, the initial classification was retained. Any discrepancies or ambiguities were reviewed in relation to the contextual information portrayed in the vignette and resolved by consensus among three members of the research team. A prespecified study-defined reference classification was established for each behavior before data collection in the main study. This classification was established by consensus, considering the contextual circumstances portrayed in each scenario and the judgments obtained during pilot testing, and was used as an analytical reference for examining agreement rather than as an externally validated standard. Participants additionally completed a questionnaire assessing realism, perceived risk for patients, and perceived legal implications for healthcare professionals using 5-point Likert scales (Appendix B).

### 2.5. Participants and Recruitment

Participants were recruited using a purposive and convenience, network-based and snowball sampling strategy aimed at including a broad range of perspectives relevant to the interpretation of unsafe healthcare behaviors. They worked in hospitals, primary care centers and nursing homes in Madrid, Alicante, Seville, Málaga, and Granada (Spain).

Two main participant groups were included: healthcare professionals and community and public representatives. Healthcare professionals included primary care professionals, hospital professionals, healthcare managers, and nursing home professionals. Community and public representatives included representatives from patient and consumer associations, journalists, social media influencers, representatives of civic organizations involved in health-related issues. This composition was intended to include individuals whose professional, advocacy, social, or community roles gave them the potential to shape or amplify societal views about healthcare, error, and professional responsibility, and thereby influence the opinions, attitudes, and perceptions of others, rather than to represent specific civil groups or society as a whole.

Sample size was estimated considering the interpretative purpose of the study, the number of participants commonly used in trigger-film studies [26], and the need to ensure sufficient representation of the main participant profiles within each group [27,28]. Eighteen principal profiles were considered according to professional role, care setting, and gender: primary care, 6 profiles (physicians, nurses, and other healthcare professionals × women/men); hospital care, 6 profiles (same structure); nursing home, 2 profiles (women/men, irrespective of professional role); healthcare managers, 2 profiles (women/men); and community/public representatives, 2 profiles (women/men). A target of approximately 25 participants per profile was set to capture variation in perspectives and reasoning, corresponding to an expected sample of approximately 450 participants.

Eligible healthcare professionals were required to have a stable professional position in primary care, hospital care, or nursing home settings. Eligible community and public representatives were adults recognized for their influence or participation in community, communication, institutional, or advocacy activities related to health or public opinion. Additional inclusion criteria for all participants included age ≥18 years, willingness to participate in group sessions, and provision of written informed consent.

Exclusion criteria included ongoing legal disputes with healthcare institutions, previous participation in formal patient safety or Just Culture committees, or personal experience of a severe adverse event that could significantly influence interpretation of the scenarios.

Recruitment was conducted through professional networks, healthcare organizations, scientific societies, patient associations, and institutional contacts. Efforts were made to ensure heterogeneity in professional background, organizational role, and gender distribution in order to capture diverse interpretative perspectives and minimize the influence of stereotyped responses.

### 2.6. Procedure

Thirty-six group sessions with healthcare professionals and thirteen sessions with community and public representatives were conducted during the study period. Some sessions involving community and public representatives were facilitated by laypersons following a citizen science approach [29].

After viewing each vignette, participants individually completed a structured response booklet in which they classified the observed behaviors as honest error, at-risk behavior, or reckless behavior and provided written explanations for each classification. Responses were completed before any group discussion took place; therefore, the classifications and written explanations analyzed in this study represented independent individual judgments rather than group-generated consensus. Once the booklets had been completed, a facilitated discussion was held to verify that participants had correctly understood the clinical situation and the behaviors depicted, thereby supporting the interpretability and validity of the written responses. These discussions were not audio-recorded, transcribed, or included in the qualitative analysis. The definitions provided to participants are available in Appendix C.

Each group session evaluated two video-based vignettes. Vignette allocation was purposively matched to participants’ professional or experiential context, so that the situations presented were sufficiently familiar and relevant to their field of practice. At the same time, allocation across sessions ensured that each vignette was evaluated by more than one group and, where appropriate, by participants with different profiles. The order of presentation was kept consistent within each session.

After viewing each vignette, participants also rated three characteristics of the scenario using 5-point Likert scales: the extent to which the situation was perceived as realistic, whether it was considered to involve a risk of an adverse event for the patient, and whether it was considered to involve legal risk for the healthcare professional. These ratings were used to describe participants’ appraisal of the plausibility and perceived relevance of the vignettes. For each item and vignette, valid responses were summarized using the mean, standard deviation, and the proportion of participants selecting ratings of 4 or 5. Participants who had not viewed a given vignette were excluded from the corresponding denominator.

### 2.7. Data Analysis

For the data analysis, the information in the response booklets was transcribed into a structured database and organized according to participant group, vignette, character, behavior, classification category, and explanatory comments. The unit of quantitative analysis was each individual classification of a specific behavior portrayed in a vignette. Because each vignette could contain several behaviors requiring classification, participants contributed more than one classification opportunity per vignette. The total number of classification opportunities therefore corresponded to the total number of behavior-level judgments expected from the vignettes viewed by the participants.

#### 2.7.1. Quantitative Analysis

Behavior classifications were summarized using frequencies and percentages overall and by participant group, video scenario, and individual behavior. For inferential analyses, each behavior-level classification was compared with the corresponding reference classification established before the main study. Exact agreement was defined as classification concordance, with each analyzable judgment coded as concordant (1) or non-concordant (0).

Factors associated with concordance were examined using a mixed-effects logistic regression model with a participant-level random intercept to account for repeated judgments within participants. Participant group was the principal predictor of interest, with gender and professional experience included as covariates. Community/public representatives, men, and participants with more than 10 years of professional experience were the reference categories. The adjusted model used complete cases for gender and professional experience; participants who preferred not to disclose either variable (gender and/or professional experience) were excluded, as was the single non-binary participant because a separate gender estimate was not feasible. Results were reported as adjusted odds ratios (ORs) with 95% confidence intervals (CIs) and two-sided *p* values. Overall effects were assessed using Wald tests, and pairwise group comparisons were adjusted using the Holm method.

Because vignette allocation was purposively matched to participants’ professional or experiential context, participant group and scenario exposure were not fully independent. Sensitivity analyses were therefore conducted among groups with overlapping vignette exposure, additionally adjusting for video scenario and, in a separate model, for individual behavior.

Finally, because the proportion of non-analyzable judgments varied across participant groups, a secondary logistic generalized estimating equations (GEE) analysis examined their association with participant group, using participant as the clustering variable and an exchangeable working correlation structure. Community/public representatives were the reference group.

#### 2.7.2. Qualitative Analysis

Participants’ written explanations constituted the qualitative dataset. For each predefined behavior, participants were required to select one of the three classification categories and to justify their choice in an open-ended response, specifying which features of the conduct they considered relevant to its classification, where applicable, and the considerations on which they based their classification. The unit of analysis was each individual written response recorded in the response booklet. These responses were analyzed using a descriptive qualitative content analysis to identify the main criteria participants used to justify their behavioral classifications. All available written explanations were included in the qualitative analysis, without subsampling.

An initial set of descriptive categories was developed inductively by three members of the research team after repeated reading of all written responses. The preliminary coding framework was based on recurrent explanatory criteria emerging from the data (e.g., intention, workload and contextual pressure, omission of safety barriers, professional responsibility and foreseeability of harm). Disagreements in coding or category assignment were discussed between the researchers and, when consensus could not be reached, were resolved by a fourth member of the research team. The resulting framework was subsequently compared with established patient safety taxonomies of contributing factors to ensure conceptual consistency, although the categories themselves remained grounded in participants’ written explanations.

Subsequently, two researchers independently coded all written responses using the agreed coding framework. Discrepancies were discussed with a third researcher until consensus was reached. The distribution and recurrent patterns of explanatory criteria within each participant group were summarized descriptively to identify common patterns of reasoning.

The researchers involved in coding had backgrounds in social and health sciences and relevant experience in patient safety and qualitative analysis. To preserve independence of judgment, the researchers who conducted the qualitative coding were not involved in selecting the final vignettes from the initial set of candidate scenarios or in establishing the prespecified reference classifications. The coding framework and analytical interpretations were grounded in the written responses collected in the main study, while pilot testing informed contextual understanding of the vignettes. Divergent or less frequent responses were also reviewed during consensus discussions.

### 2.8. Ethics

The study was approved by the Research Ethics Committees of Hospital Universitario Ramón y Cajal (reference 203/24; 23 October 2024), Hospital Universitario Fundación Alcorcón (reference 24/96; 29 October 2024), Hospital Universitari Sant Joan d’Alacant (reference 25/001; 28 January 2025), Hospital Universitario Príncipe de Asturias (reference OE 24/2025; 5 March 2025), and CEIm Provincial de Granada (reference SICEIA-2025-000681; 2 April 2025).

### 2.9. Generative AI Statement

During the preparation of this work the authors used ChatGPT, based on OpenAI’s GPT-5.5 model, in order to improve readability and language. After using this tool, the authors reviewed and edited the content as needed and take full responsibility for the content of the published article.

## 3. Results

### 3.1. Participants 

A total of 449 participants were included in the study: 318 healthcare professionals, and 131 community and public representatives. Participant characteristics are summarized in Table 1.

### 3.2. Overall Distribution of Classifications

Across all video-based vignettes, 2976 classification opportunities were recorded (Table 2). Of these, 2610 responses corresponded to classifications within the three predefined categories: honest error, at-risk behavior, or reckless behavior. The remaining 366 responses included 314 blank answers, 39 mixed classifications, or 13 responses indicating that no safety incident had occurred.

Non-analyzable judgments were unevenly distributed across participant groups. They represented 25.8% of eligible judgments among primary care professionals, compared with 7.1% among community and public representatives, 3.8% among hospital professionals, 3.4% among healthcare managers, and 0.3% among nursing home professionals. In a secondary analysis accounting for clustering of judgments within participants, participant group remained associated with the probability of a non-analyzable response (Wald χ^2^ = 81.70, df = 4; *p* < 0.001); compared with community and public representatives, primary care professionals had higher odds of providing a non-analyzable judgment (OR 4.51, 95% CI 2.71–7.53). This pattern was strongly concentrated in three behaviors from Video 4, the primary care consultation vignette: failure to review previous clinical information (57/109, 52.3%), re-prescription without reviewing the clinical history (53/109, 48.6%), and persistence with the same diagnosis despite lack of clinical improvement (59/109, 54.1%).

Overall, at-risk behavior was the most frequent classification (n = 1058), accounting for 40.5% of valid classifications, followed by honest error (n = 875; 33.5%) and reckless behavior (n = 677; 25.9%). At-risk behavior was the predominant category in all five participant groups. Nursing home professionals showed the highest proportion of at-risk behavior classifications among valid classifications (45.2%) and the largest difference between at-risk behavior and honest error (10.8 percentage points). Healthcare managers showed the most balanced distribution between honest error and at-risk behavior (38.0% and 39.0%, respectively). Community and public representatives showed the highest proportion of reckless behavior among valid classifications (29.8%), compared with 25.3% among primary care professionals, 26.1% among hospital professionals, 23.0% among healthcare managers, and 20.4% among nursing home professionals.

### 3.3. Agreement with the Reference Classification

Exact concordance with the prespecified reference classification was observed in 1264 of the 2610 classifications (48.4%) (Table 3). Concordance varied according to the reference behavioral category and was highest for reckless behavior (67.4%), compared with honest error (48.3%) and at-risk behavior (46.0%). Disagreement was therefore more pronounced for behaviors classified by reference as honest error or at-risk behavior, with classifications frequently shifting between adjacent categories.

### 3.4. Factors Associated with Concordance

Participant group was associated with concordance (Wald χ^2^(4) = 10.09, *p* = 0.039), whereas gender (*p* = 0.568) and professional experience (Wald χ^2^(4) = 5.10, *p* = 0.277) were not (Table 4). In Holm-adjusted pairwise comparisons across participant groups, nursing home professionals showed higher concordance than community/public representatives (adjusted *p* = 0.032) and primary care professionals (adjusted *p* = 0.049); the remaining contrasts were not statistically significant.

In sensitivity analyses restricted to participant groups with overlapping vignette exposure, participant group was not associated with concordance after additional adjustment for video scenario (overall *p* = 0.768) or individual behavior (overall *p* = 0.746). The effect for nursing home professionals could not be estimated independently of scenario because this group evaluated only the nursing home vignettes (Table A4).

### 3.5. Classification Patterns by Video Scenario

Classification patterns and concordance with the prespecified reference varied substantially across video scenarios (Table 5). The blood draws in primary care scenario showed the highest proportion of honest error classifications, whereas the pediatric emergency department scenario showed a relatively balanced distribution between honest error and at-risk behavior. At-risk behavior predominated in the remaining scenarios. Concordance with the reference classification was highest for medication administration in a nursing home (64.4%) and lowest for the primary care consultation (43.3%) and trauma emergency department (43.9%) scenarios.

In the medication administration in a nursing home scenario, the two behaviors showed different classification patterns. Incorrect medication preparation by the nursing assistant was most often classified as an honest error (55.9%), whereas administration of the medication to the wrong resident was predominantly classified as at-risk behavior (72.9%). Written explanations emphasized omitted verification, scope-of-practice, and supervision safeguards when interpreting medication preparation by a nursing assistant and subsequent administration to the wrong resident. In the patient transfer scenario, at-risk behavior also predominated, although concordance was lower (51.7%). Participants’ explanations highlighted the coexistence of contextual pressure with foreseeable and preventable risk, particularly in relation to inexperienced staff and insufficient supervision. Communication after the incident was more often interpreted through relational and ethical considerations.

The pediatric emergency department scenario showed a relatively balanced distribution between honest error and at-risk behavior, with concordance close to the overall average (50.5%). Incorrect prescription of a medication dose was predominantly classified as honest error, whereas omission of treatment verification was classified as at-risk behavior. Concordance was lower in the primary care consultation (43.3%) and trauma emergency department (43.9%) scenarios, where incomplete assessment, failure to review available information, and lack of reassessment generated more heterogeneous judgments across the three categories.

In the intensive care unit scenario, at-risk behavior predominated and concordance was 48.8%. Written explanations particularly emphasized omitted safety barriers and insufficient supervision of newly incorporated staff.

### 3.6. Behavior-Level Classification Patterns

Several behaviors showed relatively consistent classification patterns across participant groups (Table A3). The incorrect prescription of a medication dose by Dra. Navas was the behavior most frequently classified as honest error. Similarly, initial diagnostic focus on the main presenting complaint and sample misidentification during routine practice were predominantly classified as honest error, although some variability was observed across participants.

In contrast, behaviors involving omission of expected safety actions were particularly emphasized as at-risk behavior. This included failure to verify prescribed treatment, failure to review previous clinical information, failure to reassess new symptoms, and failure to supervise newly incorporated staff.

Behaviors perceived as clear breaches of professional responsibility showed the highest proportions of reckless behavior classifications. This included refusal to help a colleague, and failure to check patient identity.

Some behaviors showed overlap between at-risk and reckless behavior classifications, particularly when participants considered that available clinical information, supervision responsibilities, or safety procedures had been disregarded despite foreseeable risk.

### 3.7. Differences Between Participant Groups

Although all participant groups used similar conceptual distinctions between honest error, at-risk behavior, and reckless behavior, descriptive differences emerged in the relative importance assigned to contextual factors, safety barriers, professional responsibility, and relational aspects of care (Table 6).

Primary care professionals emphasized workload, time pressure, fatigue, and diagnostic uncertainty, whereas hospital professionals placed greater emphasis on procedural safeguards, verification processes, patient identification, and supervision. Healthcare managers referred to organizational accountability, leadership, and risk governance, while community and public representatives emphasized trust, empathy, attentiveness, confidentiality, and visible professional diligence. Nursing home professionals particularly highlighted medication verification, role boundaries, staff experience, resident vulnerability, and supervision. Across all groups, omission of expected safety barriers was frequently associated with at-risk behavior classifications, whereas behaviors perceived as clear breaches of professional duty or professionalism were usually classified as reckless behavior.

### 3.8. Interpretative Criteria Underlying Classifications

Participants’ comments referred to intention, contextual pressure, foreseeability of risk, availability of safety barriers, and perceived professional duty when explaining their classifications (Table 7).

Behaviors were more frequently classified as honest error when participants emphasized absence of intention, fatigue, workload, stress, multitasking, system pressure, routine practice, or human fallibility. In contrast, classifications shifted toward at-risk behavior when participants identified omission of expected safety actions, including failure to verify treatment, check patient identity, reassess symptoms, review clinical information, communicate relevant information, or supervise less experienced staff.

Reckless behavior classifications were more frequent when participants perceived a clear breach of professional responsibility, disregard of established procedures, failure to fulfil a basic duty of care, or violation of ethical or relational expectations such as confidentiality, attentiveness, or professionalism.

Overall, participants appeared to distinguish between the occurrence of human error and the omission of available safeguards intended to reduce foreseeable risk.

### 3.9. Participant Appraisal of Vignette Realism and Perceived Risk

Participants generally rated the video-based vignettes as realistic and as depicting situations with meaningful potential for patient harm (Table A5). Mean realism ratings exceeded 4.0 in six of the seven scenarios and ranged from 4.19 to 4.69 among these scenarios; 80.0–98.3% of responses were ratings of 4 or 5. Perceived patient risk was similarly high in six scenarios, with mean scores between 4.43 and 4.81 and 86.0–98.9% of responses rated 4 or 5. The blood-draw scenario received comparatively lower ratings for realism (mean 3.86, SD 1.05) and perceived patient risk (mean 3.93, SD 1.20). Perceived legal risk for the professional was more variable across scenarios, with mean scores ranging from 3.42 to 4.51. The highest legal-risk ratings were observed for the pediatric emergency department and intensive-care scenarios, whereas lower ratings were observed for the blood-draw, primary-care consultation, and nursing-home medication scenarios.

## 4. Discussion

This study explored how individuals with different perspectives on healthcare delivery, from clinical and organizational to community and lay perspectives, classified behaviors that could expose patients to risk or cause harm within a Just Culture framework. The findings suggest that participants’ explanations recurrently reflected a combination of considerations related to human fallibility, contextual pressure, foreseeability of risk, availability of safety barriers, and perceived professional duty.

An important finding was that agreement with the prespecified study-defined reference classification varied across the three behavioral categories. Overall, agreement was observed in approximately half of the judgments, but it was clearly higher for reckless behavior, for which about two thirds of classifications coincided with the reference. In contrast, at-risk behavior showed greater variability in interpretation, with judgments frequently shifting toward either honest error or reckless behavior. Thus, within the boundary-focused scenarios examined, reckless behavior showed comparatively greater agreement with the prespecified reference, whereas at-risk behavior emerged as a particularly relevant gray area between human fallibility and clearly reckless conduct.

The participants’ appraisal of the vignettes also provides additional support for their perceived plausibility and clinical relevance. Most scenarios received high ratings for realism and for the potential to result in patient harm, suggesting that the classification differences observed in the study were unlikely to be explained solely by participants regarding the situations as implausible or clinically irrelevant. The greater variability in perceived legal risk is also informative, as judgments about professional legal exposure may depend more strongly on how responsibility, procedural deviation, foreseeability, and potential consequences are interpreted. The comparatively lower ratings for the blood-draw scenario indicate that not all vignettes were perceived as equally realistic or consequential and should therefore be considered when interpreting scenario-level differences.

Across participant groups, at-risk behavior was the most frequent classification category. This prominence should be interpreted in light of the vignette design, which intentionally included behaviors situated near the boundaries between Just Culture categories, as the purpose was to examine how participants reasoned when classification was not self-evident rather than to confirm agreement in unequivocal cases. In the nursing home scenarios, participants particularly referred to verification, supervision, and scope-of-practice safeguards. However, because nursing home professionals evaluated only these scenarios, this pattern cannot be separated from vignette exposure and should not be interpreted as an independent effect of professional setting. This reinforces the importance of adapting Just Culture and incident review frameworks to care settings where resident vulnerability, staffing constraints, role boundaries and delegated tasks are central to safety [30,31].

Participants’ explanations suggested a continuum in the attribution of responsibility. Contextual factors such as workload, cognitive overload, and routine practice favored interpretations of honest error, whereas omission of feasible and expected safeguards shifted judgments toward at-risk behavior. Within the scenarios examined, at-risk classifications appeared to depend on how participants balanced contextual constraints against foreseeability, availability of safeguards, and professional responsibility. When participants perceived that a clear professional duty, ethical obligation, or established safety expectation had been disregarded, classifications were more likely to shift toward reckless behavior [32]. This pattern suggests that participants combined situational explanations with responsibility attributions when evaluating unsafe healthcare behavior, consistent with attribution theory [16,17].

Recent literature has emphasized that effective implementation of Just Culture depends on balancing learning, psychological safety, transparency, and credible accountability mechanisms rather than simply replacing blame with non-accountability, suggesting that contextual explanations do not automatically eliminate professional accountability when foreseeable safeguards are available but not used [3]. When considered alongside previous Just Culture literature, these patterns are consistent with the view that Just Culture should not be interpreted as a no-blame approach [33,34]. Rather, it requires proportionate accountability based on conduct, organizational and cultural context, foreseeability, preventability, and professional responsibility. The findings also align with emerging restorative approaches, which emphasize not only fair behavioral assessment and proportionate accountability, but also restoration of trust, inclusion of affected stakeholders, organizational learning, and relational repair following healthcare harm [14].

Differences between participant groups may offer important insights into how unsafe healthcare behaviors are socially interpreted. Although these between-group differences were not robust after adjustment for video scenario or individual behavior, the influence of vignette exposure may have made underlying stakeholder-specific tendencies more difficult to distinguish. These findings therefore underscore the importance of considering both stakeholder perspective and situational context when examining how unsafe behaviors and professional responsibility are interpreted. These findings can be viewed through the lens of Social Representations Theory, which emphasizes that judgments about health-related phenomena are not solely individual cognitive evaluations but are also shaped by socially shared meanings, professional norms, values, and practice contexts [35,36]. Recent studies in healthcare have shown that such representations can influence how professionals understand patient safety, responsibility, preventive practices, and adherence to established safeguards [37]. From this perspective, the shared use of the three Just Culture categories alongside different emphases on contextual pressure, procedural safeguards, organizational responsibility, and relational expectations may reflect partly different professional and social representations of error and accountability. The stakeholder-specific emphases observed in the written explanations should therefore be understood as descriptive interpretative patterns rather than fixed characteristics of each group. This perspective may be particularly relevant when implementing Just Culture in settings where formal patient-safety principles centered on systems learning coexist with broader professional and societal representations of error and individual responsibility conditioned by the legal framework.

### 4.1. Implications for Just Culture Practice

First, the observed variability around the at-risk category suggests that this intermediate zone deserves explicit attention in Just Culture training and incident-review frameworks. Unlike clearly reckless conduct, these situations may require reviewers to weigh contextual constraints against foreseeability of risk, availability and feasibility of safeguards, normalization of unsafe practices, and professional responsibility. Many unsafe healthcare behaviors occupied an intermediate zone in which contextual pressure helped explain the behavior but did not fully justify the omission of feasible safety actions or available safeguards [38]. This distinction may be particularly relevant in the real context during post-incident review and organizational decision-making to minimize patient safety incidents and preventable harm.

Second, the findings may also inform the use of structured deliberative approaches during incident investigation. Classification decisions should not rely exclusively on intuition, hierarchy, emotional reactions, or outcome severity. Structured review processes could support more explicit and transparent consideration of intention, foreseeability, contextual constraints, available safeguards, professional duty, supervision responsibilities, and organizational contributing conditions.

Third, although psychological safety, organizational trust, and reporting behavior were not measured in this study, the classification variability observed here may be relevant to these outcomes. Previous literature suggests that interpreting honest error as culpable conduct may discourage reporting and learning [39], whereas normalization of unsafe practices may weaken perceptions of fairness and accountability, and patient trust [40]. Effective Just Culture implementation therefore requires balancing learning-oriented responses with credible accountability mechanisms [2].

These findings are also compatible with the London Protocol 2024, particularly its emphasis on systems analysis and the assessment of defenses and barriers during incident investigation [41,42]. Structured consideration of human fallibility, omitted safeguards, foreseeable risk, and professional responsibility may complement this systems-based approach when interpreting unsafe behaviors.

Finally, the findings suggest that Just Culture may benefit from incorporating realistic case-based discussion involving multiple perspectives, including professional, managerial, and community/public perspectives. Video-based vignette approaches may offer a useful strategy for facilitating reflection on how contextual factors, omitted safeguards, ethical expectations, and professional obligations shape interpretation of unsafe healthcare behaviors [24,43].

### 4.2. Strengths and Limitations

This study has several strengths. The study included healthcare professionals, managers, and community/public representatives, providing clinical, organizational, and societal perspectives. Standardized video-based vignettes ensured that participants exposed to a given scenario evaluated the same presentation. In addition, the integration of quantitative classifications with qualitative explanations provided insight not only into how behaviors were classified, but also into the reasoning underlying those classifications.

Several limitations should also be acknowledged. Regarding study design, the use of simulated video-based vignettes cannot fully reproduce the emotional, legal, organizational, and relational complexity of real adverse events and post-incident investigations, and judgments may therefore differ from those made under real conditions. The exclusion of fatal outcomes may further limit generalizability to more severe incidents. Regarding the study materials, the comic-style presentation may have influenced participants’ perceptions of emotional realism and seriousness, this potential framing effect was not specifically assessed; the supplied Just Culture definitions may have anchored participants’ judgments toward the predefined framework; and the fixed presentation order and differences in vignette content and complexity may also have influenced responses. Because the vignettes were intentionally designed to explore tensions between the three behavioral categories, the observed concentration of judgments around category boundaries may also partly reflect this design feature. Regarding sampling and participants, the non-probability sampling strategy limits generalizability, and the heterogeneous community/public group should not be considered representative of the general population. Rather, they represent a purposively selected group of socially influential actors who may contribute to shaping or amplifying public views about healthcare, error, and professional responsibility. Because non-analyzable responses were unevenly concentrated across groups and behaviors, descriptive comparisons based only on valid three-category classifications may also be affected by selective denominator bias. Responses may also have been influenced by previous exposure to patient safety concepts, local organizational culture, or social desirability bias. Regarding vignette allocation and comparative analyses, the number of classification opportunities differed across participant groups and scenarios, and vignette allocation was purposively matched to participants’ professional or experiential context. Participant group and scenario exposure were therefore not fully independent, so differences between stakeholder groups cannot be attributed exclusively to professional or stakeholder background and may partly reflect the clinical situations evaluated. Although the mixed-effects model accounted for repeated judgments within participants, additional dependence may have arisen from participants sharing group sessions and from multiple behaviors being nested within the same clinical scenarios. These sources of clustering were not simultaneously modeled. Regarding classification responses, some behaviors, particularly in primary care scenarios, generated non-analyzable judgments, indicating that assigning a single Just Culture category was not always straightforward when contextual pressures and omitted clinical actions coexisted.

These limitations should be considered collectively when interpreting the findings. Nevertheless, the study adopted a pragmatic approach, using standardized vignettes derived from real clinical situations and evaluated by participants with relevant professional or community experience. Although no simulated design can fully reproduce the emotional, organizational, legal, and relational complexity of an actual adverse-event investigation, standardization made it possible to examine and compare judgments under consistent conditions while retaining clinically plausible contextual features. The findings should therefore be interpreted primarily as evidence of patterns of classification and reasoning elicited under these standardized but practice-informed conditions.

### 4.3. Future Research

Future studies should examine whether structured Just Culture training improves consistency in behavior classification and whether decision-support tools enhance perceptions of fairness, psychological safety, transparency, and trust during post-incident review processes. Additional research should also explore the influence of professional background, organizational culture, previous involvement in adverse events, and patient safety training on classification decisions.

Methodologically, future studies should use balanced or randomized vignette allocation and, where feasible, fully crossed or factorial designs that systematically vary contextual factors and outcome severity, while incorporating validated measures of perceived intentionality and controllability and multilevel analyses suited to the nested data structure. Vignette-based judgments should also be compared with decisions made in real or reconstructed incident investigations to assess their external validity.

Further investigation of gender-related attribution patterns may also be warranted, as the present study was not specifically designed to assess gender bias in interpretation of unsafe healthcare behaviors [44]. Including patients and family members affected by adverse events, with appropriate ethical safeguards, could additionally contribute to understanding how Just Culture decisions are perceived outside healthcare organizations [45,46].

Future research should also examine these findings in the context of open disclosure after adverse events [47,48]. The way patients, families, professionals and the wider public conceptualize error, responsibility and blame may influence how explanations are received after an incident, particularly when professionals need to communicate about honest error [2,48,49]. Understanding these social interpretations of error may help design disclosure strategies that are transparent, fair and credible, while also reducing the fear among professionals that any explanation of an incident will automatically be interpreted as an admission of individual fault [50].

## 5. Conclusions

This study suggests that judgments of unsafe healthcare behaviors reflected consideration of the interaction between human fallibility, contextual pressure, omitted safety barriers, foreseeability of risk, and perceived professional duty. Many classifications fell within the at-risk category, where contextual explanations coexisted with expectations regarding the use of feasible and available safeguards. Concordance with the prespecified reference classification was moderate and was substantially higher for reckless behavior than for honest error or at-risk behavior, showing greater variability in agreement around these category boundaries. The findings also indicate that different stakeholder groups may give different weight to contextual, procedural, organizational, and relational considerations when interpreting unsafe behavior, although future research should determine whether these patterns are reproducible across different healthcare contexts and when stakeholder groups evaluate comparable clinical situations.

These patterns may have practical implications for Just Culture implementation by highlighting the value of structured and transparent consideration of both contextual factors and professional responsibility. Incorporating multiple stakeholder perspectives into case-based reflection may also help healthcare organizations better understand how decisions concerning error, risk, and accountability are interpreted by professionals and community/public stakeholders.

## Figures and Tables

**Table 1:** Participant characteristics.

Category	n (%)
**Participant group**	
Primary care professionals	109 (24.3)
Hospital professionals	102 (22.7)
Nursing home professionals	59 (13.1)
Healthcare managers	48 (10.7)
Community and public representatives	131 (29.2)
Total	449 (100)
**Gender**	
Male	104 (23.2)
Female	284 (63.3)
Non-binary	1 (0.2)
Prefer not to disclose *	60 (13.4)
**Professional experience**	
2 to 12 months	9 (2.0)
1 to 2 years	22 (4.9)
3 to 5 years	40 (8.9)
6 to 10 years	61 (13.6)
>10 years	303 (67.5)
Prefer not to disclose *	14 (3.1)

* Response option for participants who preferred not to disclose the corresponding personal information, in accordance with applicable privacy and data protection regulations.

**Table 2:** Distribution of behavior classifications by participant group.

Participant Group	Total Opportunities	Valid Classifications	Honest Error, n (%)	At-risk Behavior, n (%)	Reckless Behavior, n (%)
Primary care professionals	1,091	810	281 (34.7)	324 (40.0)	205 (25.3)
Hospital professionals	654	629	203 (32.3)	262 (41.7)	164 (26.1)
Nursing home professionals	295	294	101 (34.4)	133 (45.2)	60 (20.4)
Healthcare managers	207	200	76 (38.0)	78 (39.0)	46 (23.0)
Community and public representatives	729	677	214 (31.6)	261 (38.6)	202 (29.8)
Total	2976	2610	875 (33.5)	1058 (40.5)	677 (25.9)

**Table 3:** Concordance with the prespecified reference classification.

Reference Classification	Judgments, n	Classified as Honest Error, n (%)	Classified as At-Risk Behavior, n (%)	Classified as Reckless Behavior, n (%)
Honest error	1125	**543 (48.3)**	415 (36.9)	167 (14.8)
At-risk behavior	1307	316 (24.2)	**601 (46.0)**	390 (29.8)
Reckless behavior	178	16 (9.0)	42 (23.6)	**120 (67.4)**
Total	2610	875 (33.5)	1058 (40.5)	677 (25.9)

Bold values on the diagonal indicate exact concordance between participant classification and the prespecified reference classification.

**Table 4:** Factors associated with concordance with the reference behavior classification.

Factor/Category	Behavior-Level Judgments, n	Concordant, n (%)	Adjusted OR (95% CI)	*p* Value
Gender				Overall *p* = 0.568
Women	1542	754 (48.9)	1.06 (0.87–1.30)	0.568
Men	679	314 (46.2)	Reference	—
Professional experience				Overall *p* = 0.277
2–12 months	61	21 (34.4)	0.61 (0.34–1.10)	0.101
1–2 years	102	46 (45.1)	0.76 (0.48–1.20)	0.239
3–5 years	193	100 (51.8)	1.15 (0.82–1.62)	0.406
6–10 years	298	150 (50.3)	0.99 (0.74–1.31)	0.921
>10 years	1567	751 (47.9)	Reference	—
Participant group				Overall *p* = 0.039
Primary care professionals	737	345 (46.8)	1.05 (0.82–1.36)	0.686
Hospital professionals	523	250 (47.8)	1.12 (0.85–1.47)	0.416
Nursing home professionals	294	167 (56.8)	1.64 (1.18–2.28)	0.003
Healthcare managers	175	87 (49.7)	1.21 (0.83–1.76)	0.328
Community/public representatives	492	219 (44.5)	Reference	—

**Table 5:** Distribution of valid classifications by video scenario.

Video Scenario	Valid Classifications, n	Honest Error, n (%)	At-Risk Behavior, n (%)	Reckless Behavior, n (%)	Concordant with Reference *, n (%)
Pediatric Emergency Department	899	350 (38.9)	331 (36.8)	218 (24.2)	454 (50.5)
Primary Care Consultation	652	179 (27.5)	282 (43.3)	191 (29.3)	282 (43.3)
Trauma Emergency Department	378	88 (23.3)	166 (43.9)	124 (32.8)	166 (43.9)
Intensive Care Unit	201	60 (29.9)	91 (45.3)	50 (24.9)	98 (48.8)
Blood Draws at the Healthcare Centre	186	97 (52.2)	55 (29.6)	34 (18.3)	97 (52.2)
Medication Administration in a Nursing Home	118	46 (39.0)	63 (53.4)	9 (7.6)	76 (64.4)
Patient Transfer in a Nursing Home	176	55 (31.2)	70 (39.8)	51 (29.0)	91 (51.7)
Total	2610	875 (33.5)	1058 (40.5)	677 (25.9)	1264 (48.4)

* Scenario-level concordance was calculated by comparing each behavior-level judgment with the prespecified reference classification for that specific behavior and then aggregating exact matches within each video scenario.

**Table 6:** Main explanatory criteria identified in participants’ written justifications according to participant group.

Dimension	Primary Care Professionals	Hospital Professionals	Healthcare Managers	Community and Public Representatives	Nursing Home Professionals
Main lens used to interpret honest error	Workload, time pressure, fatigue, clinical uncertainty	Workload and pressure, but within a procedural safety frame	System constraints, resources, interruptions, organizational conditions	Absence of intention, lack of resources, human error, good faith	Workload, routine care, staff experience, role boundaries, resident vulnerability
Main trigger for at-risk behavior	Omission of usual clinical steps; incomplete assessment; lack of checking	Failure to apply safety barriers, protocols, verification or supervision	Avoidable risk due to lack of verification, prioritization, delegation or control	Context may explain behavior but does not remove risk; insufficient checking, communication or attention	Omission of medication verification, unsafe delegation, insufficient supervision, transfer without adequate support
Main trigger for reckless behavior	Failure to fulfil a basic professional duty; lack of diligence	Clear breach of protocol or professional responsibility	Failure of role responsibility, leadership or supervision	Lack of professionalism, empathy, listening, privacy or respect	Assuming tasks beyond competence, failure to supervise vulnerable residents, disregard of basic safety procedures

**Table 7:** Recurrent explanatory criteria identified in participants’ written justifications for each behavioral classification.

Classification	Core Interpretative Criteria	Illustrative Participant Quotations	Examples from the Vignettes
Honest error	Absence of intention; human fallibility; fatigue; workload; stress; task complexity; routine slip; system pressure	“There was no intention to cause harm”; “The workload and pressure may explain what happened”; “Fatigue may have contributed to the error”; “It was a human error that could happen to anyone”; “The working conditions forced the professional to multitask”	Incorrect drug prescription under pressure; initial diagnostic focus on main complaint; sample misidentification as a punctual routine error
At-risk behavior	Omission of a safety barrier; foreseeable but not deliberate risk; incomplete checking; poor prioritization; insufficient communication; lack of reassessment or supervision	“The professional failed to verify the treatment”; “An available safety check was not performed”; “There was a foreseeable risk of losing patient follow-up”; “The patient should have been reassessed after the new symptom”; “Previous clinical information should have been reviewed”	Not checking prescribed treatment; not doing double-check; moving patient without notification; not reassessing after vomiting; not supervising new staff
Reckless behavior	Breach of a basic professional duty; clear failure of diligence; ignoring relevant information; violation of protocol; lack of professionalism; relational or ethical breach	“The professional failed to fulfil a basic duty of care”; “A clearly established procedure was disregarded”; “The behavior showed insufficient empathy toward the patient”; “The conduct was considered incompatible with expected professional standards”; “Patient confidentiality was not adequately protected”	Refusing to help a colleague; not checking identity when required; ignoring clinical deterioration; not supervising when responsible; exposing confidential information

## Data Availability

The raw data supporting the conclusions of this article will be made available by the authors on request.

## Abbreviations

The following abbreviations are used in this manuscript:

STROBE	Strengthening the Reporting of Observational Studies in Epidemiology
GROVE	Guideline for RepOrting Vignette Experiments

## Appendix A

**Table A1:** Conceptual design characteristics of the video-based vignettes.

Vignette	Setting	Main Unsafe Behavior	Key Contextual Factors	Intended Interpretative Tension
Pediatric Emergency Department	Pediatric emergency department	Incorrect dose prescription and omitted treatment verification	Workload, interruptions, multitasking, emergency pressure	Honest error vs. at-risk behavior
Primary Care Consultation	Primary care center	Failure to reassess persistent symptoms and review previous information	Time pressure, diagnostic uncertainty, fragmented follow-up	Honest error vs. at-risk behavior
Trauma Emergency Department	Emergency department	Incorrect patient identification	Overcrowding, interruptions, prioritization under pressure	At-risk behavior vs. reckless behavior
Intensive Care Unit	Intensive care unit	Medication administration confusion and lack of supervision of newly incorporated staff	High workload, complex care environment, supervision demands	At-risk behavior vs. reckless behavior
Blood Draws at the Healthcare Centre	Primary care laboratory/sample collection	Incorrect patient/sample identification	Routine practice, repetitive tasks, workload	Honest error vs. at-risk behavior
Medication Administration in a Nursing Home	Nursing home	Incorrect medication preparation	Workload, assumption of duties, lack of verification	Honest error vs. at-risk behavior
Patient Transfer in a Nursing Home	Nursing home	Fall during transfer to a wheelchair	Lack of experience, overconfidence, excessive workload, lack of supervision	At-risk behavior vs. reckless behavior

## Appendix B

**Table A2:** Assessment of the suitability of the video-based vignettes for study use.

	Participants Exposed	Realism (Mean, SD)	Perceived Risk of AE for Patients (Mean, SD)	Perceived Risk of Legal Implications for Healthcare Professionals (Mean, SD)
Pediatric Emergency Department	30	4.5 (0.8)	4.9 (0.3)	4.2 (0.8)
Primary Care Consultation	10	4.8 (0.5)	4.8 (0.5)	4.7 (0.5)
Trauma Emergency Department	18	4.8 (0.4)	4.9 (0.2)	4.6 (0.6)
Intensive Care Unit	21	4.3 (0.7)	4.9 (0.3)	4.3 (0.8)
Blood Draws at the Healthcare Centre	10	4.7 (0.8)	3.7 (1.5)	3.8 (1.5)
Medication Administration in a Nursing Home	12	4.5 (0.5)	4.7 (0.6)	4.1 (0.8)
Patient Transfer in a Nursing Home	12	4.7 (0.7)	4.7 (0.5)	4.1 (0.8)

Ratings were obtained using 5-point Likert scales ranging from 1 to 5, with higher scores indicating greater perceived realism, greater perceived patient-safety risk, and greater perceived potential for legal implications for the healthcare professional, respectively. These ratings were used to support pilot refinement of the vignettes and were not intended as measures of psychometric construct validity or discrimination between scenarios. Vignette-specific sample sizes are not mutually exclusive because pilot participants could evaluate more than one vignette. The number of valid responses could also vary across the three rating items because of missing responses.

## Appendix C

Just Culture: An organizational environment that fosters transparency, respect, and fairness. Staff feel safe expressing their opinions and learn from errors.

Honest error: An error caused by intrinsic conditions of the task, system or communication failures, pressure, workload, or equipment malfunction, among other factors.

At-risk behavior: Behavioral choices that increase risk where the risk is not recognized, is underestimated, or is mistakenly believed to be justified by contextual circumstances, routine practice, or perceived operational necessity.

Reckless behavior: Conscious disregard of a substantial and unjustifiable risk, representing a significant deviation from expected professional standards or safety practices.

## Appendix D

**Table A3:** Distribution of classifications by behavior.

Vignette	Character	Behavior	Valid Classifications, n	Honest Error, n (%)	At-Risk Behavior, n (%)	Reckless Behavior, n (%)
Pediatric Emergency Department	Dr. Navas (physician)	Incorrect prescription of drug dose	190	145 (76.3)	39 (20.5)	6 (3.2)
		Does not check prescribed treatment	176	47 (26.7)	94 (53.4)	35 (19.9)
		Multitasks	172	73 (42.4)	80 (46.5)	19 (11.0)
	Andrea García (nurse)	Administration of an incorrect medication dose	183	69 (37.7)	76 (41.5)	38 (20.8)
	Dr. Navarro (physician)	Does not help colleague	178	16 (9.0)	42 (23.6)	120 (67.4)
Primary Care Consultation	Dr. Sánchez (physician)	Focuses on hip pain rather than abdominal pain	137	88 (64.2)	40 (29.2)	9 (6.6)
	Dr. Molina (physician)	Focuses on test results and orders another test	134	45 (33.6)	74 (55.2)	15 (11.2)
		Does not consult previous clinical information	83	7 (8.4)	42 (50.6)	34 (41.0)
	Dr. Riquelme (physician)	Bases the assessment only on test results and does not inquire about additional symptoms	132	19 (14.4)	54 (40.9)	59 (44.7)
		Does not review history and prescribes NSAIDs again	88	8 (9.1)	45 (51.1)	35 (39.8)
		Maintains the same diagnosis despite no change in the patient’s clinical presentation	78	12 (15.4)	27 (34.6)	39 (50.0)
Trauma Emergency Department	Lucas Valdés (nurse)	Takes the patient out of the clinical workflow without notification	103	38 (36.9)	42 (40.8)	23 (22.3)
		Does not reassess patient after vomiting	84	4 (4.8)	53 (63.1)	27 (32.1)
	Dr. Gutiérrez (physician)	Does not check patient identity	101	16 (15.8)	38 (37.6)	47 (46.5)
	Fernando Sánchez (orderly)	Addresses the patient by name and reason for visit	90	30 (33.3)	33 (36.7)	27 (30.0)
Intensive Care Unit	Carlota Ulla (nurse)	Incorrect medication administration	101	45 (44.6)	38 (37.6)	18 (17.8)
	Elisa Martínez (head nurse)	Does not supervise newly incorporated professional	100	15 (15.0)	53 (53.0)	32 (32.0)
Blood Draws at the Healthcare Centre	Elisa Reche (nurse)	Incorrect sample identification	98	53 (54.1)	27 (27.6)	18 (18.4)
	Luis Ibáñez (nurse)	Incorrect sample identification	88	44 (50.0)	28 (31.8)	16 (18.2)
Medication Administration in a Nursing Home	Ana (nursing assistant)	Incorrect medication preparation	59	33 (55.9)	20 (33.9)	6 (10.2)
	Inés (nurse)	Medication administration to wrong resident	59	13 (22.0)	43 (72.9)	3 (5.1)
Patient Transfer in a Nursing Home	Laura (nursing assistant)	Unsafe resident transfer without sufficient experience	59	7 (11.9)	29 (49.2)	23 (39.0)
	Marta (nurse)	Insufficient supervision of inexperienced staff	59	14 (23.7)	28 (47.5)	17 (28.8)
	Dr. Morales (physician)	Inadequate communication after incident	58	34 (58.6)	13 (22.4)	11 (19.0)

## Appendix E

**Table A4:** Sensitivity analysis of the concordance model.

Model	Primary Care vs. Community/Public	Hospital vs. Community/Public	Healthcare Managers vs. Community/Public	Overall Group *p*
Adjusted for scenario	1.15 (0.88–1.51)	1.06 (0.79–1.43)	1.09 (0.73–1.63)	0.768
Adjusted for individual behavior	1.17 (0.88–1.57)	1.07 (0.78–1.48)	1.12 (0.72–1.73)	0.746

Sensitivity analyses were restricted to participant groups with overlapping vignette exposure. Nursing home professionals were not included because this group evaluated only nursing home scenarios, making participant-group and scenario/behavior effects non-identifiable separately.

## Appendix F

**Table A5:** Participants’ appraisal of vignette realism and perceived patient and professional legal risk in the main study.

Video Scenario	Realism Mean ± SD; % 4–5	Perceived Patient Risk Mean ± SD; % 4–5	Perceived Legal Risk Mean ± SD; % 4–5
Pediatric Emergency Department	4.68 ± 0.58; 96.2	4.78 ± 0.46; 98.9	4.51 ± 0.72; 89.7
Primary Care Consultation	4.51 ± 0.75; 92.0	4.43 ± 0.76; 86.0	3.78 ± 1.24; 65.8
Trauma Emergency Department	4.19 ± 0.89; 80.0	4.56 ± 0.68; 91.5	4.35 ± 0.83; 81.9
Intensive Care Unit	4.43 ± 0.70; 90.4	4.72 ± 0.52; 96.8	4.50 ± 0.73; 92.4
Blood Draws at the Healthcare Centre	3.86 ± 1.05; 61.4	3.93 ± 1.20; 70.5	3.42 ± 1.26; 52.3
Medication Administration in a Nursing Home	4.45 ± 0.90; 87.9	4.52 ± 0.63; 93.1	3.90 ± 1.09; 65.5
Patient Transfer in a Nursing Home	4.69 ± 0.57; 98.3	4.81 ± 0.47; 96.6	4.32 ± 0.90; 83.1
